# Estimating of Bending Force and Curvature of the Bending Plate in a Three-Roller Bending System Using Finite Element Simulation and Analytical Modeling

**DOI:** 10.3390/ma14051204

**Published:** 2021-03-04

**Authors:** Ionel Gavrilescu, Doina Boazu, Felicia Stan

**Affiliations:** 1 Department of Mechanical Engineering, Dunarea de Jos University of Galati, Domneasca 47, 800008 Galati, Romania; ionel.gavrilescu@ugal.ro (I.G.); doina.doazu@ugal.ro (D.B.); 2 Center of Excellence Polymer Processing, Dunarea de Jos University of Galati, Domneasca 47, 800008 Galati, Romania

**Keywords:** three-rolling bending process, finite element analysis, bending force, vertical displacement

## Abstract

Many industries such as shipbuilding require steel bending plates in a wide range of radii, thus bending machines are often designed and produced on a custom basis in shipyards. From a design perspective, however, the bending force and the radius of the bending plate as a function of vertical displacement of the upper roller must be known. In this paper, a hybrid numerical–analytical approach is proposed to investigate the three-roller bending process for two plates of steel used in the naval industry. Firstly, the bending process is modeled using the finite element (FE) method and regression models for the bending force as a function of plate thickness and vertical displacement of the upper roller were constructed. Then, based on the findings from FE analysis, using the bent bar theory, two analytical expressions for the bending force were derived. Using geometric and deformation compatibilities, analytical expressions for the vertical displacement of the upper roller as a function of the curvature of the bending plate were also developed. The FE results suggest that the cross section of the plate is practically a plastic hinge in the tangent area of the upper roller and that the deformation compatibilities must be considered in order to estimate the curvature radius of the bending plate using analytical formulations. These results are of practical importance in designing rolling machines to estimate the setting parameters.

## 1. Introduction

Cylindrical and conical steel shells are important components used in various engineering applications such as bilge planking as a part of the naval hull, cylindrical tanks, pressure vessels, etc. [1]. Rolling machines with three or four rollers are generally considered for manufacturing shells (bending plates) with different curvatures [1,2,3,4]. In order to design a rolling machine, it is necessary to calculate the bending force and the curvature radius of the bending plate for given material properties and geometrical parameters. Therefore, efforts have been devoted to the prediction of the bending force or curvature radius by experimental, theoretical, and numerical approaches.

In general, the bending force is estimated using an analytical approach based on the one-dimensional beam theory combined with experimental analysis for validation. For example, Chudasama and Raval established an analytical model for the prediction of the bending force for the three-roller conical multi-pass bending process [4]. Furthermore, Chudasama and Raval proposed an analytical model for force prediction in the three-roller conical bending process, taking into account the effects of various material properties and geometrical parameters [5], which can also be used to estimate the roller plate-interface friction. Padgan et al. have conducted an analytical and experimental analysis of bending forces for five materials, such as aluminum alloy, copper alloy, stainless steel, gray cast iron, and magnesium alloy [6]. However, the analytical models are not able to fully describe the complex deformation behavior of the materials during the bending process due to oversimplified assumptions [2,7]. Therefore, in the attempt to optimize the roll bending process, in the last years, the finite element method (FEM) was applied to simulate the bending process and extract different information, such as bending force, the radius of curvature, stress-strain state in the contact area under various conditions, and the finite element (FE) environment [8,9,10,11,12,13]. For example, Tran investigated the forming process of an asymmetrical roll bending machine using a three-dimensional (3D) dynamic FE model in the Ansys/LS-DYNA environment and the results were validated by experiments [8]. Feng and Champliaud reported on the numerical simulation of cylindrical roll bending to predict the position of the lateral roll [9] and on the conical roll bending [10] using Ansys/LS-DYNA environment with explicit time integration. Ktari et al. carried out two-dimensional (2D) modeling of the rolling process in three roll bending pyramid systems using Abaqus explicit module [11]. Taylor et al. investigated the influence of various parameters of the three-roll bending process on the final radius of curvature of the bent sheet using an elastoplastic explicit dynamic FE method under the LS-DYNA environment [12]. Neto et al. developed an FE model to analyze the stress–strain in the vicinity of the contact area where the plastic deformation increases due to the forming tool [13]. Moreover, several attempts have been made to combine the FE simulation with the analytical modeling of different bending systems. Patel et al. proposed a moment curvature-based model for elastoplastic micro-beam bending to solve a micro-cantilever subjected to a normal load undergoing large deflection [14]. Pandit and Srinivasan presented an explicit numerical approach for three-point bending of a thin elastoplastic beam undergoing large deflection supported on cylindrical rollers with a radius comparable to the deflection [15]. Using both analytical and finite element approaches, Zemin et al. studied the three-roll bending of a large workpiece using a model based on one dimension beam theory to determine the inner radius displacement and the Abaqus FE model for optimizing the process parameters [16]. It was shown that, in general, the FE analysis provides more accurate results for the bending force and radius of the curvature compared with the experimental data than the oversimplified analytical models based on bending beam theory.

In summary, even though a number of researches have been carried out to improve the roll bending technology, estimation of the bending force and the curvature radius of the bending plates as a function of the displacement of the upper roller is still difficult at this stage, especially for large sheets and mainly relays on expensive physical experiments. Thus, the three-roller bending process needs further investigation both numerically and analytically. Moreover, the FE analysis, in combination with existing analytical theories, may offer a cheap and easy alternative to the trial-and-error, experimental approach.

The goal of the paper is to derive simple yet effective analytical expressions for calculating the bending force and the vertical displacement of the upper roller as a function of the radius of curvature, taking into account the findings from the FE analysis of the three-roller bending process. The novelty of this paper consists of coupling the plastic hinge condition observed during the FE analysis with the bent bar theory.

## 2. Numerical Simulation of the Three-Roller Banding Process

### 2.1. Research Methodology

**Figure 1:**
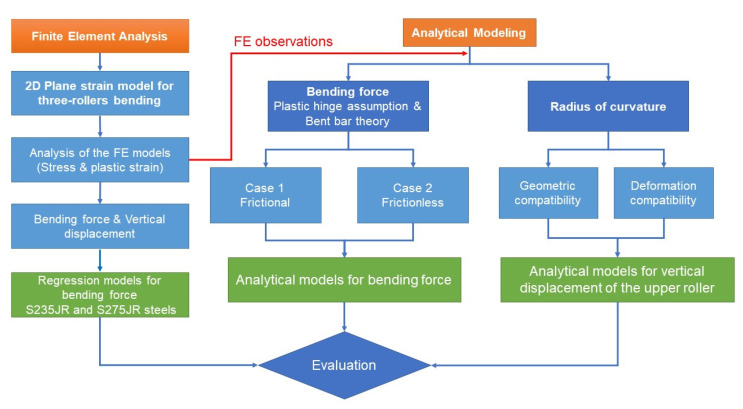
Flow chart for the hybrid numerical–analytical approach.

Considering the complexity of the deformation during the rolling process, a hybrid numerical–analytical approach is proposed to investigate the three-roller bending process, as shown in Figure 1. First, the three-roller bending process is modeled using the FE method and Ansys Workbench Static Structural program (version 19.0, 2019, Ansys, Inc., Canonsburg, PA, USA), under plane strain conditions. In addition, regression models for the bending force as a function of plate thickness and vertical displacement of the upper roller are derived for two types of structural steel commonly used in the shipbuilding industry. Second, based on the findings from FE analysis, according to which the cross section of the plate is practically a plastic hinge in the tangent area of the upper roller, two analytical approaches for estimating the bending force in different hypotheses (frictional and frictionless) were derived based on the bent bar theory and compared with the FE results. Third, using geometric and deformation compatibilities, analytical expressions for the vertical displacement of the upper roller as a function of the radius of curvature, by taking into account the thickness of the sheet, were derived and verified against the FE results.

### 2.2. Finite Element Model

**Figure 2:**
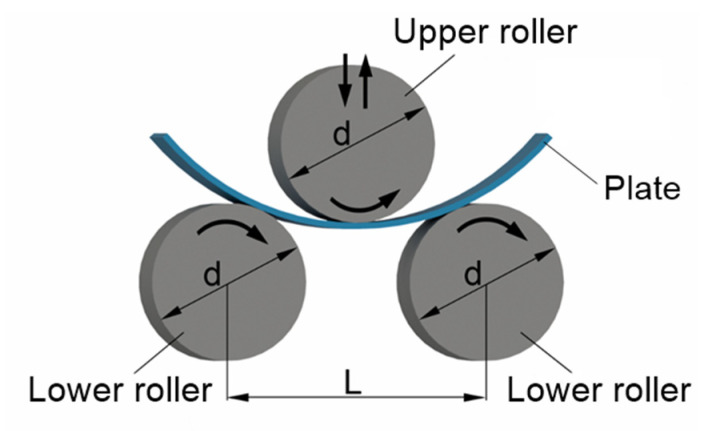
Schematic representation of the three-roller bending process (d—the outer diameter of the rollers, L—the distance between the lower rollers).

Figure 2 shows a schematic representation of the three-roller bending process with identical cylindrical rollers. This system employs one upper roller and two lower rollers as a forming tool. The radius of curvature of the bending plate is controlled by changing the vertical position of the upper roller. To reach a desire (final) curvature radius, the upper roller moves down in a vertical direction pressing down the plate.

Three design parameters, i.e., the vertical displacement of the upper roller, the force applied to the upper roller, and the torsional moments at the axes of the rollers, are required for design a three-roller bending system according to the imposed specifications, such as the plate thickness, final radius of the bending plate, and material properties.

To determine the design parameters for the three-roller bending system, in this paper, a static finite element analysis was carried out using the Ansys Workbench Static Structural program [17] with specific nonlinear settings. It should be pointed out that modeling large plastic deformations, which is the case of the three-rolling process, is based on the use of logarithmic deformations (Hencky), the Jaumann derivative of the Cauchy stress tensor, and the von Mises flow condition [17].

The bending system, particularly used in the naval shipyards, consists of three identical rollers with an outer diameter of 189 mm and a distance between the centers of the lower rollers of 320 mm. A previous study [18] indicated that, for the displacements of the upper roller equal to the roller radius, the applied force increases asymptotically and, therefore, the moments at the axes of rollers increase asymptotically, indicating that the technological solution may be inappropriate for the specified conditions.

To simulate the deformation behaviors, because the analysis of the entire three-roller bending process is very complicated, a two-dimensional (2D) model was considered. Because the plate’s width to thickness ratio is more than 200, and with the assumption of uniform loading along the width of the plate, the plane strain model is suitable. The simulation process was broken down into three stages as a function of time (as a pseudo-variable), which are (i) stage 1: The time varies in the range of 0–1 s in which the upper roller has an imposed vertical downward displacement; (ii) stage 2: The time varies in the range of 1–2 s in which the rollers have an imposed rotation; and (iii) stage 3: The time varies in the range of 2–3 s in which the upper roller has an imposed vertical upward displacement.

**Figure 3:**
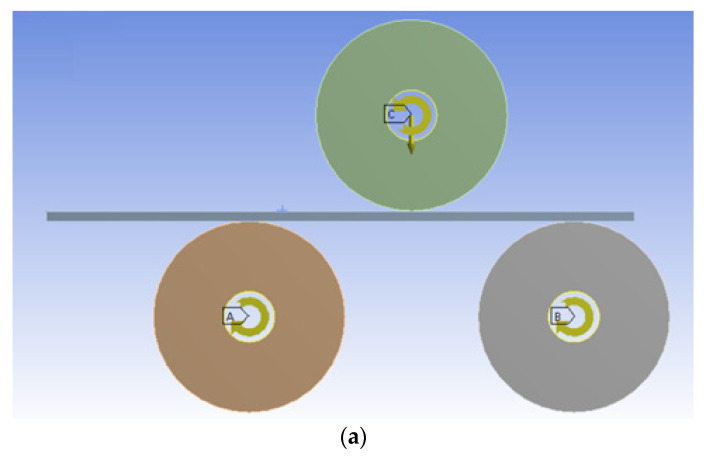

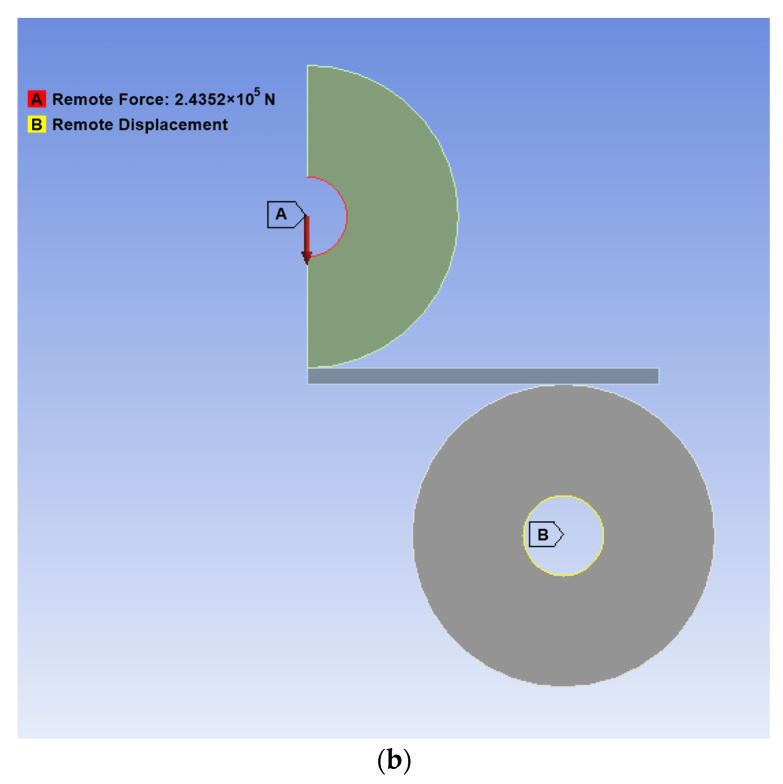
(**a**) A two-dimensional (2D) model for the three-roller bending process for stages 1 and 2 and (**b**) a 2D symmetric model for stage 1.

Figure 3a shows the 2D plane strain model for the three-roller bending process corresponding to simulation stages 1 and 2. In addition, a symmetrical 2D plane strain model corresponding to stage 1 (i.e., the vertical displacement of the upper roller) was developed to gain information that could be used in the analytical approach (on a symmetrical model) for the bending force calculation (Figure 3b). In this particular case, the center of the bottom roller was fixed, and the force is applied in the center of the upper roller along the axis of symmetry.

**Figure 4:**
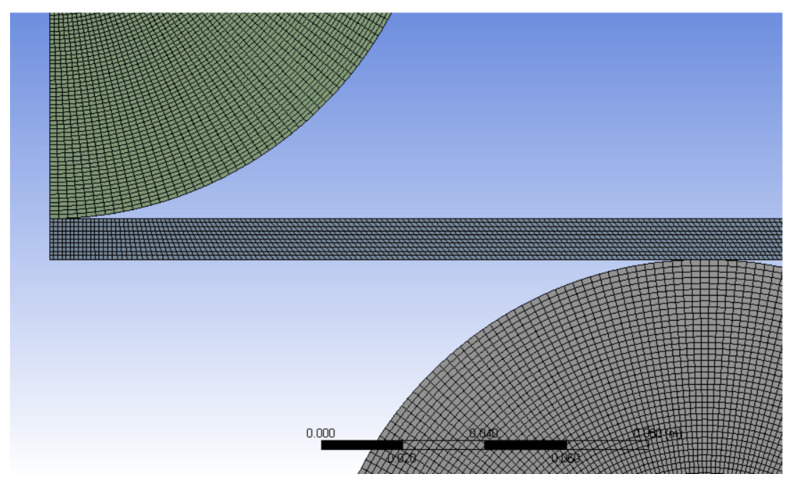
Discretization of the three-roller bending model.

The models were drawn in the xy plane using the Design Modeler (version 19.0, 2019, Ansys, Inc., Canonsburg, PA, USA) included in the Static Structural module [17]. The origin of the reference system is in the center of the upper roller, whereas the x and y axes define the plane (in plane strain), where x is the horizontal axis and y is the vertical axis. The plate was discretized with 2D eight-node quadrilateral higher order elements (type PLANE 183 (Ansys, Inc., Canonsburg, PA, USA) [17], with large deflection and contact conditions included. These elements account for the nonlinearities introduced by the nonlinear behavior of the material, large displacements, and the use of contact elements between the roller surfaces and the plate [17]. The finite element size was 1 mm, resulting in 10 elements with that thickness for the 10-mm plate thickness in Figure 4. A total number of 15,000 elements were used to model the plate.

The contact between the plate and the rollers was considered frictional in the sense of Coulomb with a coefficient of friction of 0.3. In the 2D model, for the surface-to-surface contact, the “contact” and “target” elements are CONTA192 (Ansys, Inc., Canonsburg, PA, USA) and TARGE169 (Ansys, Inc., Canonsburg, PA, USA) for contact bodies and target bodies, respectively. The augmented Lagrangian formulation with asymmetric behavior was considered and the small sliding was considered off. The model worked without activating the interface treatment Adjust to Touch [17].

The simulation of the bending process was performed by increasing the vertical displacement of the upper roller from 0 to the maximum physical displacement imposed by the geometry of the system (see Table 1). However, to avoid numerical instability (i.e., the applied force increases asymptotically with increasing the radius of curvature [18]), the vertical displacement was limited to 50 mm. The simulations were carried out for two types of structural naval steels, for which an elastoplastic material model with isotropic hardening was considered, as in Table 1. It should be noted that the spring-back of the bending plate was not taken into account, and the rollers were assumed to be deformable in the linear-elastic domain.

### 2.3. Numerical Simulation Results

**Figure 5:**
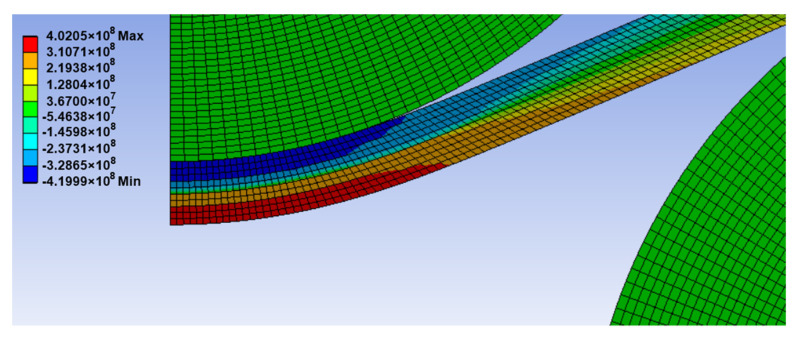
Distribution of the normal stress $\sigma_{X}$ (in Pa) for the 10-mm plate thickness (S235JR steel).

Figure 5 shows the variation of the normal stress $\sigma_{X}$ for the 10-mm plate thickness of S235JR steel. The results illustrate that the normal stress in each point of the cross section in the tangent area of the upper roller is equal to the yield strength.

It should be noted that the normal stress, $\sigma_{X}$, is guided along the Ox-axis that is oriented along the neutral deformed surface of the plate, i.e., in each section, the Ox axis is “parallel” with the faces of the deformed plate. Furthermore, it was found that the distribution of stresses on a cross section depends on the mesh size when the calculation is in the elastoplastic domain.

**Figure 6:**
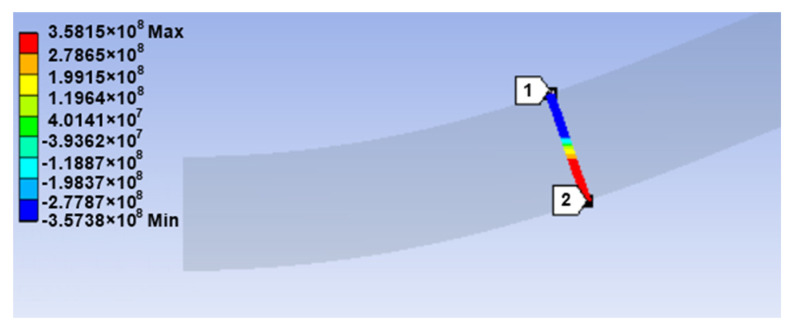
Distribution of the normal stress $\sigma_{X}$ (in Pa) in the tangent area of the upper roller (S275JR steel, 10-mm plate thickness).

The distribution of normal stress along the cross section in the tangent area of the upper roll is shown in Figure 6 for the S275JR steel. As can be seen, practically, the entire cross section of the plate is yielded, indicating that the deformation state corresponds to the “plastic hinge.”

**Figure 7:**
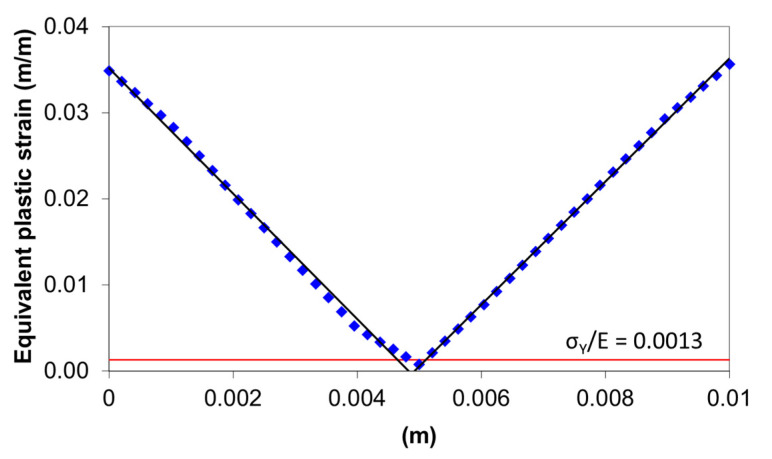
Equivalent plastic strain in the tangent area of the upper roller for the 1-mm plate thickness (S275JR steel).

Figure 7 shows the variation of the equivalent plastic strain in the cross section of the 10-mm plate in the tangent area of the upper roller. As can be seen, for almost the entire cross section, the plastic strain is greater than the $\sigma_{Y}$/E ratio, which is 0.0013 for the S275JR steel.

**Figure 8:**
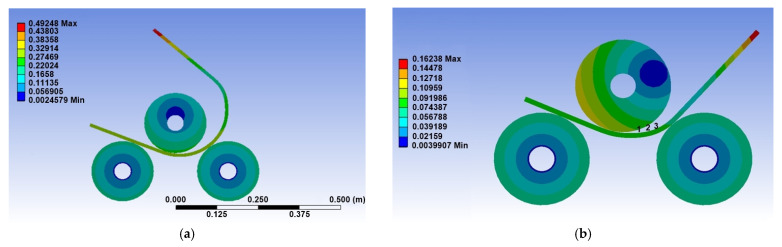
(**a**) Total deformation (in m) at the end of stage 2 and (**b**) location of the points for the estimation of the radius for the 10-mm plate thickness of S275JR steel.

Figure 8a shows the deformation of the 10-mm plate thickness at the end of stage 2. In order to estimate the final radius of the bending plate, the displacement of three points located on the inner surface of the plate was monitored during the FE analysis, as shown in Figure 8b. These points are approximately located on a circle with center (x,y) and radius *r*.

By using the least-square method, the center and the radius were determined by minimizing the sum

(1) $$S\left(x,y,r\right)=\sum_{i=1}^{3}{\left(r-r_{i}\right)}^{2},$$

in which $r_{i}$, i=1, 2, 3, is the radius corresponding to each point in Figure 8b, i.e., the distance from the coordinates of each point $\left(x_{i},y_{i}\right)$ to the center (x,y),

(2) $$r_{i}=\sqrt{{\left(x-x_{i}\right)}^{2}+{\left(y-y_{i}\right)}^{2}}.$$

**Figure 9:**
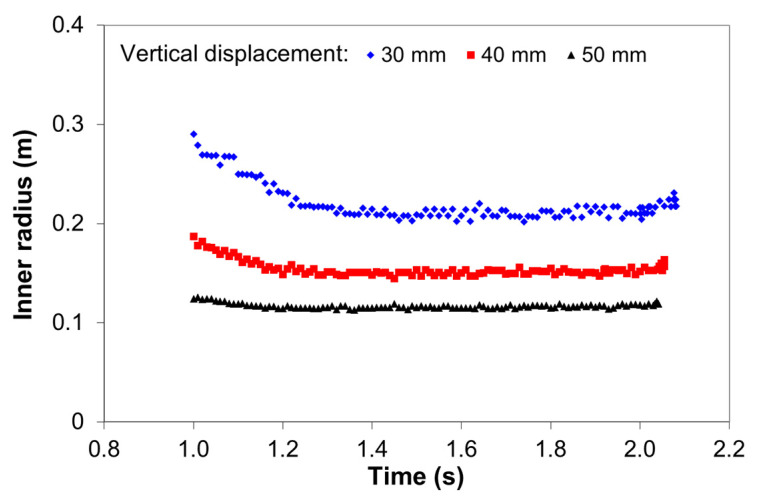
Variation of the inner radius of the bending plate as a function of time for different vertical displacements (10-mm plate thickness and S275JR steel).

Figure 9 shows the variation of the inner radius of the plate as a function of time for three vertical displacements. It can be seen that the inner radius decreases with increasing vertical displacement and tends to stabilize with increasing vertical displacement.

An approximate value for the inner radius related to a certain vertical displacement can be obtained by averaging the data over time. Therefore, the inner radius of the bending plate was found to be 218.6 ± 4.8 mm, 158.4 ± 2.9 mm, and 117.6 ± 1.5 mm for 30 mm, 40 mm, and 50 mm vertical displacement, respectively.

## 3. Analytical Modeling of the Three-Roller Bending Process

### 3.1. Radius of the Bending Plate

**Figure 10:**
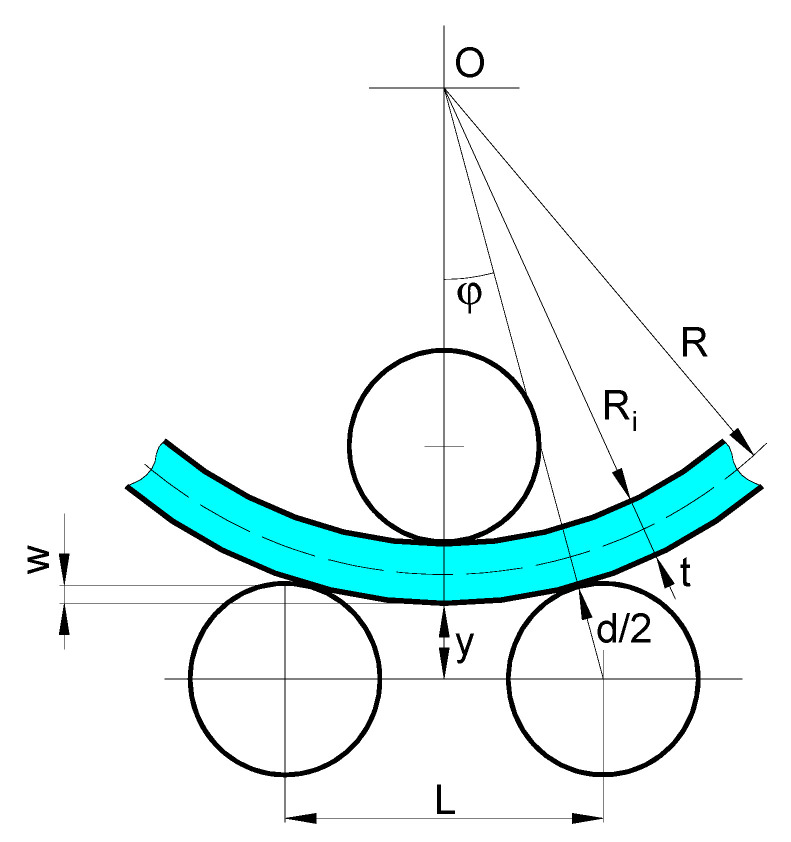
Schematic representation of the geometric compatibilities of the three-roller bending system.

The radius of curvature of the bending plate (i.e., the radius of the neutral layer) can be determined based on the geometric compatibility of the three-roller bending system, shown in Figure 10, as follows:

(3) $$R=R_{i}+\frac{t}{2},$$

where $R_{i}$ is the inner radius of the bending plate, and *t* is the plate thickness.

By invoking the geometric compatibilities of the bending system, taking into account that $sin\phi^{2}+cos\phi^{2}=1$, the following relation can be derived:

(4) $${\left(\frac{\frac{L}{2}}{R_{i}+t+\frac{d}{2}}\right)}^{2}+{\left(\frac{R_{i}+t+y}{R_{i}+t+\frac{d}{2}}\right)}^{2}=1,$$

where *y* is the distance between the horizontal axis of the lower rollers to the deformed outer layer of the plate and *d* is the diameter of the rollers.

After some mathematical manipulation and rearranging, the distance *y* reduces to

(5) $$y=\sqrt{1-{\left(\frac{\frac{L}{2}}{R_{i}+t+\frac{d}{2}}\right)}^{2}}\left(R_{i}+t+\frac{d}{2}\right)-R_{i}-t.$$

Based on Figure 10, the vertical displacement, *w*, of the upper roller can be written as

(6) $$w=\frac{d}{2}-y.$$

Substituting Equation (5) in Equation (6), the final expression for the vertical displacement as a function of the inner radius of the bending plate can be written as

(7) $$w=\frac{d}{2}-\sqrt{1-{\left(\frac{\frac{L}{2}}{R_{i}+t+\frac{d}{2}}\right)}^{2}}\left(R_{i}+t+\frac{d}{2}\right)+R_{i}+t.$$

**Figure 11:**
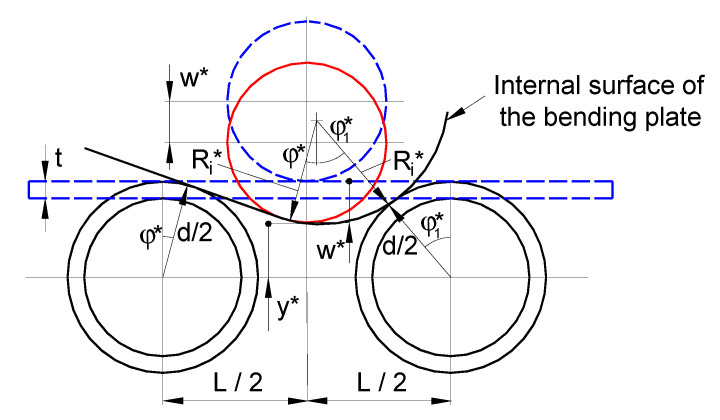
Schematic representation of the deformation compatibilities of the three-roller bending system based on FE observations.

The analytical model as in Equation (7), obtained only from geometrical compatibility and without taking into account the deformation mode of the plate or the spring-back, overestimates the inner radius of the bending plate as compared with the values predicted by the FE analysis. For the vertical displacement of 30 mm, 40 mm, and 50 mm, the relative error between the inner radius calculated by Equation (7) and FE values is 34%, 33%, and 33%, respectively. It should be pointed out that the greater the radius of the deformation, the greater the effect of the spring back. Thus, taking into account the deformation behavior from the FE analysis, which is similar to the deformation behavior of the plate during the bending process presented in [19], a new approach was proposed for calculating the inner radius as a function of the vertical displacement, as illustrated in Figure 11. It should be noted that the parameters marked by “*” stand for the correction according to the finding from the FE analysis.

Based on the new framework described in Figure 11, taking into account that ${\left(sin\phi_{1}^{*}\right)}^{2}+{\left(cos\phi_{1}^{*}\right)}^{2}=1$, the following relation can be derived:

(8) $${\left[\frac{\frac{L}{2}-\left(R_{i}^{*}-\frac{d}{2}\right)sin\phi *}{R_{i}^{*}+t+\frac{d}{2}}\right]}^{2}+{\left[\frac{y*-\frac{d}{2}+\left(R_{i}^{*}-\frac{d}{2}\right)cos\phi *}{R_{i}^{*}+t+\frac{d}{2}}\right]}^{2}=1,$$

where

(9) $$cos\phi *=\frac{\frac{d}{2}}{\frac{d}{2}+t},\;sin\phi *=\sqrt{1-{\left(\frac{\frac{d}{2}}{\frac{d}{2}+t}\right)}^{2}}.$$

After some mathematical manipulation and rearranging, the expression for y* takes the form

(10) $$y*=\sqrt{1-{\left(\frac{\frac{L}{2}-\left(R_{i}^{*}-\frac{d}{2}\right)sin\phi^{*}}{R_{i}^{*}+t+\frac{d}{2}}\right)}^{2}}\left(R_{i}+t+\frac{d}{2}\right)-\frac{d}{2}-\left(R_{i}^{*}-\frac{d}{2}\right)cos\phi^{*}.$$

As before, the vertical displacement is

(11) $$w*=\frac{d}{2}-y*.$$

Substituting Equation (10) into Equation (11), in term of the inner radius, following some manipulation of the resulting expression, the vertical displacement can be written as

(12) $$w*=d-\sqrt{1-{\left(\frac{\frac{L}{2}-\left(R_{i}^{*}-\frac{d}{2}\right)sin\phi^{*}}{R_{i}^{*}+t+\frac{d}{2}}\right)}^{2}}\left(R_{i}^{*}+t+\frac{d}{2}\right)+\left(R_{i}^{*}-\frac{d}{2}\right)cos\phi^{*}.$$

Equation (12) allows for a more accurate prediction of the inner radius of the bending plate as a function of vertical displacement.

**Figure 12:**
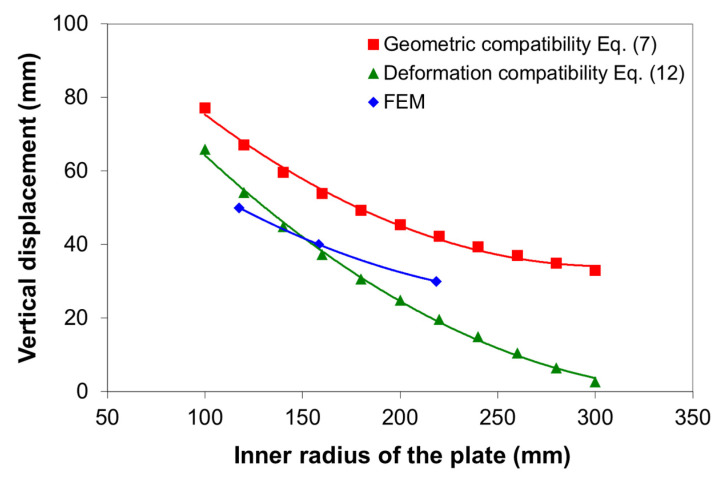
Vertical displacement of the upper roller as a function of the inner radius of the bending plate with 10 mm thickness (S275JR steel).

Figure 12 compares the vertical displacement as a function of the inner radius of the bending plate corresponding to the geometric compatibility (as in Equation (7)), the deformation compatibility (as in Equation (12)), and the FE results, for the 10-mm plate thickness. As can be seen, the geometric compatibility model overestimates the vertical displacement, as compared with the deformation compatibility model, especially at the higher inner radius in which the effect of the spring-back is very important. The relative errors between the geometric model and deformation model are 45%, 35%, and 25% for 30 mm, 40 mm, and 50 mm vertical displacement, respectively. It can be seen that the higher the inner radius is, the higher the relative error. On the other hand, the vertical displacement of the upper roller can be predicted by Equation (12) relatively well. Compared with the FE results, the relative errors of the deformation compatibility model are 19%, 2%, and 12% for 30 mm, 40 mm, and 50 mm vertical displacement, respectively.

### 3.2. Bending Force

For estimating the bending force, in this study, the findings from the FE analysis were considered, namely, (i) the normal stress for each point of the cross section reaches the yield strength in the tangent area of the upper roller and (ii) the bending deformations of the plate beyond the tangent area are small. Therefore, in order to estimate the bending force, two scenarios were considered as given below.

#### 3.2.1. Case 1 Scenario (C_1_)

The assumptions are as follows: (i) the plate has a finite thickness and width; (ii) the friction plays an important role in the three-roller bending process, i.e., the coefficient of friction between the rollers and plate is taken as 0.3; and (iii) the bending moment $M_{p}$ in the cross section of the plate situated in the tangent area of the upper roller corresponds to the so-called plastic hinge condition, i.e., the normal stress for each point of the cross-section becomes equal to the yield strength. Therefore, the bending moment can be expressed by

(13) $$M_{p}=\sigma_{Y}\frac{l\;\times \;t^{2}}{4},$$

where $\sigma_{Y}$ is the yielding strength, *l* is the width of the plate, and *t* is the thickness of the plate.

**Figure 13:**
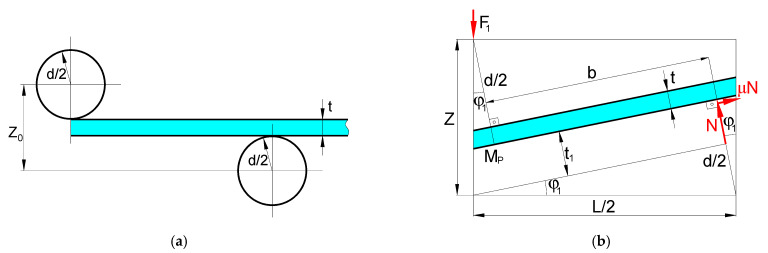
Configuration of the three-roller bending system for Case 1: (**a**) initial position of the rollers and undeformed plate and (**b**) position of the rollers and the plate at a given time and the diagram of forces applied on the plate.

The initial configuration of the three-roller bending system is given in Figure 13a, while the configuration at a given time is presented in Figure 13b. From the notations given in Figure 13b, it follows that

(14) $$t_{1}=z\;\times \;cos\phi_{1}-\frac{d}{2}-t,$$

and

(15) $$\frac{L}{2}sin\phi_{1}+zcos\phi_{1}-d-t=0,$$

taking into account that

(16) $$tan\alpha_{1}=\frac{z}{\frac{L}{2}}.$$

Based on Equations (15) and (16), following some manipulation, it can be shown that

(17) $$\phi_{1}+\alpha_{1}=arcsin\left[\frac{d+t}{\frac{L}{2}}\;\times \;cos\alpha_{1}\right].$$

Furthermore, with the geometry of Figure 13b, the function b is given by

(18) $$b=\frac{L}{2}cos\phi_{1}-zsin\phi_{1},$$

By taking into account the forces represented in Figure 13b, the bending moment $M_{p}$, which corresponds to reaching the “plastic hinge” condition in the cross section, can be written as

(19) $$M_{p}=\sigma_{Y}\frac{l\;\times \;t^{2}}{4}=N\;\times \;b,$$

considering the balance of forces in the vertical direction (in the y-axis direction), which implies that

(20) $$F_{1}=Ncos\phi_{1}+\mu Nsin\phi_{1},$$

and the bending force is simply

(21) $$F=2\;\times \;F_{1}.$$

#### 3.2.2. Case 2 Scenario (C_2_)

**Figure 14:**
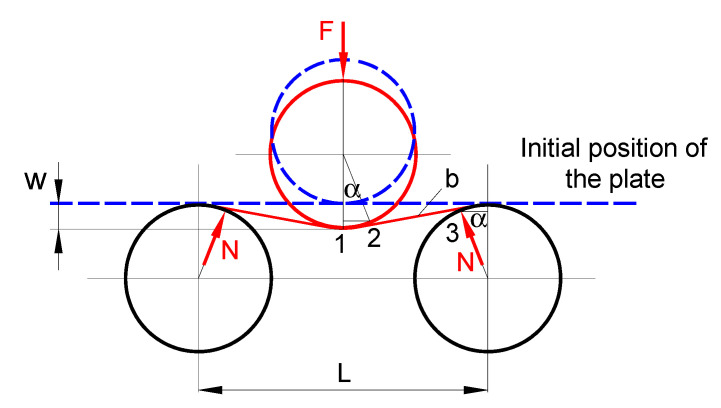
Schematic representation of the three-roller bending system and the forces applied to the sheet for Case 2.

The assumptions are as follows: (i) for estimating the deformed position of the plate, the thickness of the plate is negligible; (ii) the effect of friction is also negligible; (iii) the bending moment (Figure 14) corresponds to the high condition of plastic bending (i.e., the normal stress for each point of the cross-section is equal to the yield strength) and is given by Equation (13); and (iv) the moment in Section 3 is zero. It should be noted that, in Figure 14, the dotted lines represent the undeformed plate, while the solid line denotes the final position of the upper roller.

Considering the equilibrium of the forces that act on the three-roller bending system in Figure 14,

(22) $$N\;\times \;b=\sigma_{Y}\frac{l\;\times \;t^{2}}{4},$$

the reaction force *N* is given by

(23) $$N=\sigma_{Y}\frac{l\;\times \;t^{2}}{4}\frac{1}{b},$$

where *b* is the arm of the reaction force, and $\sigma_{Y}$ is the yield strength.

In the present context, the bending force can be calculated by

(24) F=2 × Ncosα.

Assuming that the vertical displacement of the upper roller can be approximated by

(25) $$\frac{L}{2}-2\frac{d}{2}\alpha =w,$$

then, the angle *α* (in radians) is

(26) $$\alpha =\frac{\frac{L}{2}-w}{d}.$$

Considering the three-roller bending system in Figure 14, the geometrical consideration leads to

(27) $$2\frac{d}{2}sin\alpha +bcos\alpha =\frac{L}{2},$$

and the arm *b* of the reaction force is given by

(28) $$b=\frac{1}{cos\alpha }\left[\frac{L}{2}-dsin\alpha \right].$$

Substituting Equation (28) into Equation (23), the reaction force *N* becomes

(29) $$N=\sigma_{Y}\frac{l\;\times \;t^{2}}{4}\frac{cos\alpha }{\left[\frac{L}{2}-dsin\alpha \right]}.$$

The expression for *N* is then substituted into Equation (24) to obtain the bending force in the following form:

(30) $$F=2\sigma_{Y}\frac{l\;\times \;t^{2}}{4}\frac{{cos}^{2}\alpha }{\left[\frac{L}{2}\;-\;dsin\alpha \right]}.$$

## 4. Predictive Models for Bending Force

**Figure 15:**
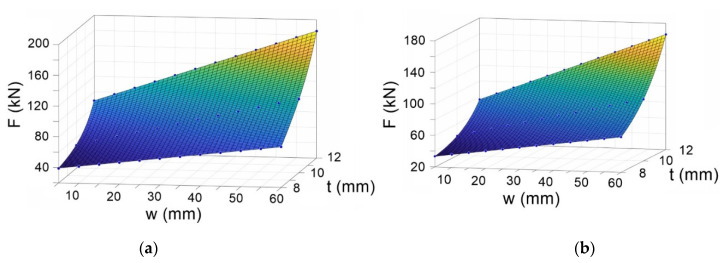
The bending force from FE analysis for (**a**) S235JR steel and (**b**) S275JR steel.

In order to determine a predictive model for the bending force, FE simulations were carried out for different geometrical and material properties, as shown in Table 1. The FE simulations were performed with the model described in Section 2 for vertical displacement of the upper roller varying from 5 mm to 78 mm. The FE results for the bending force as a function of vertical displacement of the upper roller and the sheet thickness are shown in Figure 15 for S235JR and S275JR steels.

The results obtained from the FE simulations were used to determine a predictive model for the bending force as a function of two variables, i.e., vertical displacement of the upper roller and plate thickness. The FE results were fitted using Matlab curve fitting toolbox (version R2018a, The MathWorks, Inc., Natick, MA, USA) [20,21].

The regression models for the bending force (in Newton) for the two plates of steel are expressed by the following equations:

(31) F(w,t)=25.96−0.06384w+0.0542 exp(0.001454+0.5549t)+0.5711 w exp(−1.852+0.2448t)

for the S235JR steel, and

(32) F(w,t)=33.56−22.08w+0.008891 exp(−0.002553+0.7136t)+1.123w exp(2.909+0.0128t)

for the S275JR steel.

The accuracy of the regression models was analyzed through the coefficient of determination (R^2^). The R^2^ coefficient for the S235JR and S275JR is 1.0% and 0.999%, respectively, indicating a very good correlation between the FE results and regression models. In addition, the adequacy of the derived models was also analyzed using the root-mean-square error (RMSE). The RMSE values for the S235JR and S275JR are 4.06 × 10^−5^% and 4.786 × 10^−1^%, respectively. These results show that the regression models are reliable and can be used to calculate the bending force.

**Figure 16:**
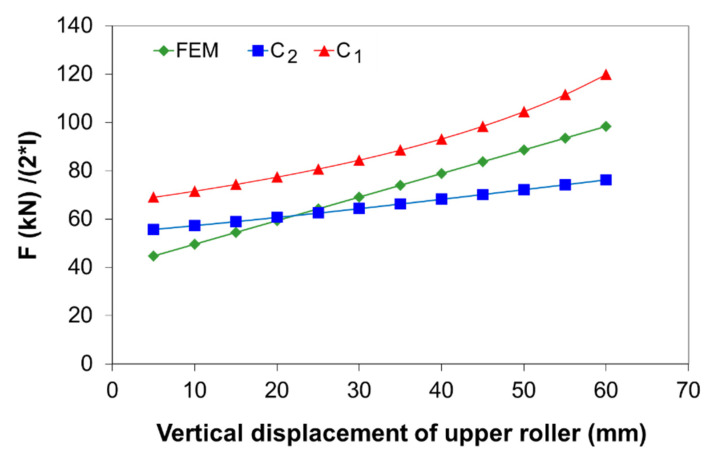
Bending force versus vertical displacement predicted based on the analytical models and the FE analysis for a 10-mm plate thickness and S235JR steel.

Figure 16 compares the bending force as a function of vertical displacement of the upper roller for the two analytical models and the FE regression model (as in Equation (31)) for a plate thickness of 10 mm and S235JR steel. It can be seen that the C_1_ analytical model (frictional, as in Equation (21)) over predicts the bending force as compared with the C_2_ analytical model (frictionless, as in Equation (30)) and FE model. The relative errors for bending forces between the C_1_ and C_2_ models vary between 19% and 36%, and the higher the vertical displacement, the greater the relative error.

Compared with the FE regression model, the average relative error, the maximum relative error, and the RMSE for the C_1_ analytical model are 27%, 54%, and 18.2%, respectively, while the average relative error, the maximum relative error, and RMSE for the C_2_ model are 13.5%, 25%, and 12%, respectively. It should be noted that the maximum relative error of 54% for the C_1_ model and 25% for the C_2_ model occurs at 5 mm vertical displacement. For the C_1_ model, it appears that the presence of friction in the analytical formulation has a noticeable effect on the deformation behavior; thus, it predicts higher values as compared with the C_2_ model. However, the bending force values calculated based on the C_2_ model are in better agreement with the FE values than those calculated by the C_1_ model. Therefore, it can be concluded that the plastic hinge condition without friction is adequate to estimate the bending force with reasonable accuracy.

## 5. Conclusions

In this study, a hybrid approach based on the finite element (FE) analysis and analytical modeling of the three-roller bending process was proposed in order to predict the bending force and the curvature radius of the bending plate. The simulation of the three-roller bending process was carried out using the Ansys Workbench Static Structural program, under plane strain conditions. Starting from the observations following the FE analysis, analytical approaches for estimating the bending force and vertical displacement of the upper roller were derived. Based on the FE analysis and the analytical modeling, the following main findings and conclusions can be drawn:

(i) A 2D FE model was established to analyze the three-roller bending process for the S235JR and S275JR steels used in the naval industry. For the system under consideration, the FE results suggest that the cross section of the plate is practically a plastic hinge in the tangent area of the upper roller;

(ii) Taking into consideration the assumption of reaching the “plastic hinge” condition in the cross section of the plate, two analytical models for the bending force were derived based on the bent bar theory. These models can be used to first-hand estimate the bending force independently of FE analysis;

(iii) Using geometric and deformation compatibilities, simple analytical models for the vertical displacement of the upper roller as a function of the curvature radius of the plate were developed and verified against the FE results. However, in order to estimate the curvature radius of the bending plate using the analytical formulation, the deformation compatibilities must be considered;

(iv) FE-based regression models for estimation of the bending force for the S235JR and S275JR steels were formulated. The models take into consideration the plate thickness (8–12 mm) and vertical displacement of the upper roller (up to 78 mm). For other values of the yielding stress, the bending force can be obtained by interpolation (because the three-roller bending system has a diameter of 189 mm and the distance between the axis of the lower rollers of the system is 320 mm).

These results are of practical importance for the industry to estimate the setting parameters required in designing a three-roller bending machine. However, future research will address the experimental validation of the derived models.

## Figures and Tables

**Table 1:** Material properties and model parameters.

Parameters	S235JR	S275JR
Yield strength (MPa)	235	275
Young modulus (Pa)	2.1 × 10^11^	2.1 × 10^11^
Tangent modulus (Pa)	2.1 × 10^9^	2.1 × 10^9^
Poisson ratio (-)	0.3	0.3
Sheet thickness (mm)	8, 10, 12	8, 10, 12
Maximum vertical displacement (mm)	50	50

## Data Availability

The data presented in this study are available on request from the corresponding author.

## References

[1] Roggendorff S., Haeusler J. (1979). Plate bending: Three rolls and four rolls compared. Weld. Met. Fabr..

[2] Hua M., Lin Y.H. (1999). Large deflection analysis of elastoplastic plate in steady continuous four-roll bending process. Int. J. Mech. Sci..

[3] Shin J.G., Lee J.H., Kim Y.I., Yim H. (2001). Mechanics-based determination of the center roller displacement in three-roll bending for smoothly curved rectangular plates. J. Mech. Sci. Technol..

[4] Chudasama M.K., Raval H.K. (2012). Analytical model for prediction of force during 3 Roller multipass conical bending and its experimental verification. Int. J. Mech. Eng. Robot. Res..

[5] Chudasama M.K., Rava H.K. (2013). An approximate bending force prediction for 3-roller conical bending process. Int J. Mater. Form..

[6] Padgan N.P., Deshpande P.D., Sakhale C.N. (2015). Force Analysis of Metal Sheet in Bending Operation on Sheet Bending Machine. Int J. Eng. Res. Tech..

[7] Jadhav C.S., Talmale P.S. (2017). Verification and Analysis of Stress-Strain Curve of Sheet Metal by using Three Roller Bending Machine. Int. J. Eng. Dev. Res..

[8] Tran H.Q. (2014). Asymmetrical Roll Bending Process Study: Dynamic Finite Element Modeling and Experiments. Ph.D. Thesis.

[9] Feng Z., Champliaud H. (2011). Modeling and simulation of asymmetrical three-roll bending process. Simul. Model. Pract. Theory.

[10] Feng Z., Champliaud H. (2011). Three stage process for improving roll bending quality. Simul. Model. Pract. Theory.

[11] Ktari A., Antar Z., Haddar N., Elleuch K. (2012). Modeling and computation of the three-roller bending process of steel sheets. J. Mech. Sci. Technol..

[12] Taylor V.K., Gandhim A.H., Moliya R.D., Raval H.K. (2008). Finite element analysis of deformed geometry in three-roller plate bending process. Proceedings of the ASME 2008 International Manufacturing Science and Engineering Conference.

[13] Neto D.M., Martins J.M.P., Oliveira M.C., Menezes L.F., Alves J.L. (2016). Evaluation of strain and stress states in the single point incremental forming process. Int J. Adv. Manuf. Tech..

[14] Patel B.N., Pandit D., Srinivasan S.M. (2017). Large elaso-plastic deflection of micro-beams using strain gradient plasticity theory. Procedia Eng..

[15] Pandit D., Srinivasan S.M. (2017). Large Elasto-Plastic Deflection of Thin Beams with Roller Support Contact, 11th International Symposium on Plasticity and Impact Mechanics. Procedia Eng..

[16] Fu Z., Tian X., Hu B., Yao X. (2013). Analytical modeling and numerical simulation for three-roll bending forming of sheet metal. Int. J. Adv. Manuf. Technol..

[17] (2018). Ansys Workbench 2018, Version 19.

[18] Gavrilescu I., Boazu D. (2017). Simulation of roll bending with three rollers pyramid system using FEM. Ann. Univ. Dunarea de Jos of Galati Fascicle VI.

[19] Hardt D.E., Constantine E., Wright A. (1992). A model of a sequential bending process for manufacturing simulation. J. Eng. Ind. Trans. ASME.

[20] (2018). MATLAB 2018, Version R2018a.

[21] Kamiński K., Solecka M. (2013). Optimization of the truss-type structures using the generalized perturbation-based Stochastic Finite Element Method. Finite Elem. Anal. Des..

